# Antibacterial Activity of *Thymus Syriacus* Boiss Essential Oil and Its Components against Some Syrian Gram-Negative Bacteria Isolates

**Published:** 2013-06

**Authors:** Ayman Al-Mariri, Ghayath Swied, Adnan Oda, Laila Al Hallab

**Affiliations:** 1 Department of Molecular Biology and Biotechnology, Atomic Energy Commission, Damascus, Syria;; 2 Department of Chemistry, Atomic Energy Commission, Damascus, Syria

**Keywords:** Essential oils, Gram-negative bacteria, Minimum inhibitory concentration

## Abstract

**Background:** Despite the medical discoveries of different medicines and advanced ways of treatment, statistics have shown that the number of patients is increasing. This may be due to chemical drugs used in healthcare, agriculture, and diets. This soaring demand in medicines urges us to look for natural sources such as aromatic plants and essential oils, which are rich in efficient compounds.

**Methods: **Extraction of essential oils was performed using a Clevenger-type apparatus. Identification was achieved using the GC-FID technique. Confirmation was made using the GC-MS technique, and isolation was done using a preparative HPLC, equipped with an aliquots collector. The microdilution broth susceptibility assay was utilized to determine minimum inhibitory concentrations (MICs).

**Results: **Our *in vitro* study demonstrated the antibacterial activity of the *Thymus syriacus* Boiss essential oil and its components against the tested isolates at levels between 0.375 and 50 µl/ml. The main components of the *T. syriacus* essential oil were carvacrol, γ-terpinene, and ß–caryophyllene. MIC_90_ values for the *T. syriacus* essential oil against the gram-negative organisms varied between 3.125 and 12.5 µl/ml. The most effective components against the gram-negative bacteria were thymol, carvacrol, dihydro-carvon, and linalool respectively.

**Conclusions: **The *T. syriacus* essential oil and some of its components exhibited very good inhibitory effects against Syrian gram-negative isolates.

## Introduction

Safety testing on essential oils, when used as directed, shows very few bad side effects or risks. Some essential oils have been approved as ingredients in food and are classified and generally recognized as safe.^1^ Essential oils should be regarded as one of the several available feed additives that have been demonstrated to have antibacterial activity against undesirable pathogenic bacteria such as *Salmonella* spp.^2^ Essential oils consist of a number of active compounds, some of them comprising more than 60 individual components that can inhibit the growth of certain microorganisms.^3^ Besides flavoring, aromatic plants have been drawn upon for their medicinal properties for centuries.^4^ As natural products with well-documented and repeatedly demonstrated efficiency against a wide range of microorganisms, essential oils receive particular attention as agents suitable for prophylactic and medical treatment.^5^ Many essential oil isolates exhibit inhibitory properties in challenge tests against microorganisms.^6^ Herbs have been found to possess antimicrobial activity and anti-viral properties.^7^

The genus *Thymus* (*Lamiaceae*) consists of more than 300 evergreen species of herbaceous perennials and sub shrubs, native to Southern Europe and Asia.^8^ This genus is represented by 38 species and altogether 64 taxa.^9^ The *Thymus* genus species (*Lamiaceae*) are well known in Syria, where their common name is Zattar.^10^ They are native plants and can be found wildly or cultivated in most Syrian provinces, especially in the north-west, coastal, and south-west regions. Five species of *Thymus* are found in Syria. *Thymus* *syriacus* Boiss are used as herbal tea and condiments. Fresh leaves are used for aromatization of home-made jams, candies, and similar confections. *Thymus* *syriacus* Boiss is also known to have positive results for coughs and other respiratory complaints, as well as some cases of gastrointestinal disorders (personal communication with local people). 

Although such anecdotal evidence exists, scientific research about the aspects of the therapeutic use of *T. syriacus* Boiss or its chemical inventory remains scarce and inconsistent. *Thymus* species are used as medicinal and aromatic plants, as well as in cosmetics and perfumery.^11^ Most aspects of their medicinal uses are related to the essential oil, which contains various levels of thymol and/or carvacrol, phenolic derivatives with strong and wide-spectrum antimicrobial activity.^12^ Species such as *T. vulgaris *L., *T. zygis Loefl *L., and *T. serpyllum* L. are the biological sources of herbal drugs Thymi herba, Thymi aetheroleum, and Serpylli herba, officially recognized in many modern pharmacopoeias such as European Pharmacopoeia 6.0.^13^

The chemical composition of essential oils is variable. For example, the concentrations of the main components of the thyme essential oil (thymol and carvacrol) can range from 3–60% of the total essential oil.^14^ Major components can constitute up to 85% of the essential oil, whereas other components are present only as a trace;^15^ nevertheless, they are also very important. The primary components are the major active ingredients, while the secondary components act synergistically to increase the total effectiveness.^16^

The antimicrobial properties of plant volatile oils and their constituents from a wide variety of plants have been assessed^17^ and reviewed.^18^ The mechanisms of action may vary greatly and depend mainly on the composition of the essential oil.^19^ The effect of essential oils can be enhanced through synergistic effects both between individual essential oils and by combination with other feed additives.^20^ The light thyme essential oil, particularly when enhanced by agar stabilizer, may be effective in reducing the number or preventing the growth of *E. coli *O157:H7 in foods.^21^ The aim of this investigation was to assess the antimicrobial activity of the *T. syriacus* Boiss essential oil and to determine its chemical composition.

## Materials and Methods

*Collection and Preparation of Plant Materials*

Leaves of *T. syriacus* were collected from three locations which differ in altitudes, climates, and rain falls, during the flowering season. The samples were cleaned from any strange plants, dust, or any other contaminants. The collected plants were air dried and were cut to pieces. The characteristics of the collection locations are presented in table 1.

**Table 1:** Collection locations and main ecological factors of *T. syriacus*

**Location name**	**Longitude**	**Latitude**	**Altitude (m)**	**Precipitation average (mm/year)**	**Highest temperature** **average (°C)**	**Lowest temperature average (°C)**
Mishtaia	36° 16'	34° 36'	400	300	32.4	9.5
Mkalis	36° 22'	34° 48'	850	450	31.6	9.3
Mountain Faleh	36°25'	34° 51'	1000	650	29.4	6.3

*Essential Oil Extraction*

Extraction of essential oils was conducted using a water steam distillation device (Clevenger-type apparatus) according to the manufacturer’s instructions.^13^^,^^22^ The device was attached to a condenser and cold water recycler (hydrodistillation technique). Distilled water was added (1:10 v/v), and each sample was distilled for 2 h. The supernatant contained essential oil, which was dehydrated by filtering through anhydrous Na_2_SO_4_. The essential oil was prepared and collected in airtight vials and stored in refrigerator.

*Identification and Isolation of the Main Components of the Essential Oils*

The identification of each pure component was accomplished by the GC-FID technique.^8^ GC analysis was carried out using a 30-m column HP-5 (0.25 mm i.d 0.4 μm film thickness) with helium as carrier gas. The oven temperature was kept at 50°C for 2 min, programmed to 110°C at a rate of 2°C/min, and kept constant for 3 min. Subsequently, it was programmed to 175°C at a rate of 4°C/min, kept constant for 2 min, programmed to 250°C at a rate of 5°C/min, and kept constant for 5 min. The injection mode was Splitless, the injector temperature was 250°C, and the detector temperature was 275°C. 

Chromatograms of the essential oils were computed by the normalization method from the GC peak areas, calculated as the mean values of two injections. Confirmation of the components of the essential oils was performed using the GC-MS technique, and isolation was conducted using a preparative HPLC (Jasco), equipped with a UV/VIS detector and aliquots collector. (The solvents were purchased from Merck [Germany].) GC-MS conditions were comprised of a mass range of 36 Amu-300 Amu, sample rate of 65, and source temperature of 260°C. The HPLC analytical conditions were optimized to have the best separation conditions and to avoid any adjacent peaks. The best HPLC separation conditions were seen as follows: mobile phase of THF/CAN.; mobile phase flow rate of 1.3 ml/min; sample volume of 150 μl; analysis time of 90 min; and detector conditions of response=fast, range=0.32. 

*Microorganisms and Growth Conditions*

Local isolates of *Escherichia coli* O157, *Salmonella typhimurium*, *Klebsiella pneumoniae*, *Yersinia enterocolitica* O9, *Brucella melitensis*, *Pseudomonas aeruginosa*, and *Proteus* spp. were grown for 24-48 h in 2YT agar (peptone, 16 g/liter; yeast extract, 10 g/liter; NaCl, 5 g/liter; and agar, 13 g/liter [Difco, BD, Spars, MD]).^23^ The bacteria were suspended in a sterile phosphate-buffered saline (PBS). Bacteria abundance in PBS was monitored by recording the optical density (OD) at 590 nm. The exact counts were assessed retrospectively by viable counts on 2YT agar plates.

*Determination of Minimum Inhibitory Concentration*

The microdilution broth susceptibility assay was employed.^24^ Three replicates of serial dilutions of the essential oils and their components were prepared in an LB broth medium in 96-well microtiter plates, using a range of concentrations for each essential oil and its components from 0.375 to 50 µl/ml. Also, 100 μl of freshly grown bacteria standardized 10^6^ CFU/ml in the LB broth were added to each well. Positive control was done with the same conditions but without essential oils, and negative control was also done with the same conditions but without adding the bacteria. The plate was incubated with shaking for 24 h at 37°C. The lowest concentration that completely inhibited visual growth was recorded and interpreted as the minimum inhibitory concentration (MIC). 

*Statistical Method*

A mean value for each test was obtained by averaging the triplicate values after log conversion. 

## Results

Table 2 shows the percentages of the essential oils from the three Syrian locations. The average concentration of the three locations was 2.08%. In addition, table 3 reveals the percentages of the compositions of the *T. syriacus* essential oil.

**Table 2:** Percentages of the essential oils in dried samples from the collection locations

**Location name**	**EO %**
Mishtaia	2.12
Mkalis	1.99
Mountain Faleh	2.09

Table 3 illustrates that the main component of the *T. syriacus* essential oil was carvacrol (36.73%), whereas the other major components were γ-terpinene (8.97%), ß–caryophyllene (6.17%), farnesol (6.07%), ocimene (4.83%), thymol (4.00%), menthol (3.40%), myrcine (3.03%), and α-pinene (2.40%). On the basis of the primary screening results (table 4), the *T. syriacus *essential oil was effective against the gram-negative bacteria isolates. MIC_90_ values for the* T. syriacus* essential oil against the *E. coli *O:157, *Y. enterocolitica* O9, *B. melitensis, Proteus* spp., *P. aeruginosa*, *S. typhimurium*, and *K. pneumoniae* isolates were 12.5, 6.25, 6.25, 3.125, 3.125, 6.25, and 3.125 µl/ml, respectively. On the other hand, the most effective components against the gram-negative bacteria were thymol (MIC_90_: from <0.375 to 1.5 µl/ml), carvacrol (MIC_90_: from <0.375 to 6.25 µl/ml), dihydro-carvon (MIC_90_: from <3.125 to 25 µl/ml), and linalool (MIC_90_: from <6.25 to 25 µl/ml), respectively.

**Table 3:** Percentages of the main components of the *T. syriacus* essential oil

**Compound**	**Location 1**	**Location 2**	**Location 3**	**Average %**
a-Pinine	2.2	2.3	2.7	2.40
Camphene	2.6	2.2	4.4	3.07
Myrcene	3.1	3.1	2.9	3.03
Limonene	2.4	2.4	1.2	2.00
o-Cymene	5.9	4.7	3.9	4.83
Cineole	4.6	2.6	2.4	3.20
γ - Terpinene	9.7	9.4	7.8	8.97
Linalool	3.3	1.9	2.9	2.70
Terpinene-4-ol	2.8	3.7	1.2	2.57
Menthol	4.1	3.2	2.9	3.40
Dihydro-carvon	3.4	4.9	4.2	4.17
Thymol	4.1	3.6	4.3	4.00
Farnesol	5.9	6.5	5.8	6.07
Carvacrol	33.4	37.3	39.5	36.73
ß -Caryophyllene	6.3	5.3	6.9	6.17
Total	93.8	93.1	94	

**Table 4:** Antimicrobial activity of the *T. syriacus* essential oil and some of its main components against some gram-negative isolates

**Essential oil and** **Components**	***E. coli*** **O157**	***Y. enterocolitica *** **O9**	***B. melitensis***	***Proteus***	***P. aeruginosa***	***S. typhimurium***	***K. neumoniae***
EO	12.5	6.25	6.25	3.125	3.125	6.25	3.125
α-Pinine	NIE	NIE	NIE	NIE	NIE	NIE	NIE
Camphene	NIE	NIE	NIE	NIE	NIE	NIE	NIE
Myrcene	NIE	NIE	NIE	NIE	NIE	NIE	NIE
Limonene	NIE	NIE	50	NIE	NIE	NIE	NIE
o-Cymene	NIE	NIE	NIE	NIE	NIE	NIE	NIE
Cineole	NIE	50	50	50	50	50	6.25
γ -Terpinene	NIE	NIE	NIE	NIE	NIE	NIE	50
Linalool	25	25	6.25	6.25	12.5	12.5	6.25
Terpinene-4-ol	NIE	50	12.5	6.25	25	6.25	12.5
Menthol	NIE	NIE	50	NIE	NIE	NIE	NIE
Dihydro-carvon	25	12.5	3.125	6.25	6.25	12.5	25
Thymol	1.5	0.375	0.75	1.5	1.5	<0.375	1.5
Farnesol	NIE	NIE	50	NIE	NIE	NIE	NIE
Carvacrol	<0.375	0.75	<0.375	<0.375	6.25	<0.375	3.125
ß -Caryophyllene	25	50	50	50	12.5	50	6.25

NIE, non-inhibitory effect

## Discussion

In recent years, more attention has been given to the plants of the *Lamiaceae* family, especially the genus *Thymus* spp. In 1979, it was reported that the average of essential oils in the *Thymus* spp dry aerial part was 2.0%,^25^ which agrees with that found in our study (2.08%). Cluster analysis of the thyme essential oils allowed the classification into three main groups: a carvacrol and thymol group (Group I) with rich oils and major antimicrobial activities, a linalyl acetate and (E)-nerolidol group (Group II), and a γ-terpinene and p-cymene group (Group III) or even sesquiterpene hydrocarbons-rich oils, showing lower antimicrobial activities than the former group.^26^ The results of our study showed that the main component of the *T. syriacus* essential oil was carvacrol (36.73%), followed by γ-terpinene (8.97%), ocimene (4.83%), menthol (3.40%), myrcine (3.03%), ß–caryophyllene (6.17%), and α-pinene (2.40%), while the average of thymol was 4%. This result did not agree with that reported by Azaz et al.^8^ who found that thymol (36.9%-56.6%) was the main component in the oils of *T. zygioides *var. lycaonicus, *T. longicaulis *subsp. chaubardii var. chaubardii (chemotype I and II) and carvacrol (60%) was the main component in the oils of *T. longicaulis *subsp. longicaulis var. subisophyllus. In addition, Baser et al.^27^ found that the essential oil of *T. zygioides* var. lycaonicus contained thymol (42.0%-57.0%) and γ-terpinene (19.5%). The percentages of the components of the essential oils in our collected plants varied among the populations according to their grown appurtenance and climate deviation; these variations were not remarkable when compared to the significant deviation observed by Burt,^21^ who reported that the *T. vulgaris *essential oil contained carvacrol (2-11%) and thymol (10-64%). In addition, Nickavar et al.^28^ reported that the main components of Iranian *T. daenensis* were thymol (74.7%), p-cymene (6.5%), ß-caryophyllene (3.8%), and carvacrol (3.6%). Miguel et al.^29^ reported that the main component of the *T. caespititius *essential oil was a-terpineol (32%). Sarikurkcu et al.^30^ reported that the essential oil composition of *T. longicaulis *was c-terpinene, thymol, and p-cymene (27.80, 27.65, and 19.38%), respectively. Nevertheless, our results more or less agree with those found by Bounatirou et al.^31^ who reported that the main components of the Tunisian *T. capitatus *Hoff. and Link. essential oils were carvacrol (62-83%), p-cymene (5-17%), c-terpinene (2-14%), and b-caryophyllene (1-4%). In another study, the essential oil of *T. longicaulis *subsp. longicaulis var. subisophyllus was reported to contain thymol (3.0%), borneol (16.0%), and p-cymene (15.0%) as the main constituents.^32^ In addition, Nejad et al.^33^ reported that the main components of a composition of the *T. caramanicus* (an endemic species grown in Iran) essential oil were carvacrol (58.9-68.9%), p-cymene (3.0-8.9%), c-terpinene (4.3-8.0%), thymol (2.4-6.0%), and borneol (2.3-4.0%). Salgueiro et al.^34^ demonstrated that the essential oils of *Thymus* *xmourae* and *T. lotocephalus*, two endemic taxa from Portugal, have the following five components: linalool, 1,8-cineole, linalool/1,8-cineole, linalyl acetate/linalool, and geranyl acetate. In this study, the *T. syriacus* essential oil compound showed very important activities against gram-negative isolates. These activities varied from 3.125 µl/ml against *Proteus* spp and *P. aeruginosa* to 12.5 µl/ml against *E. coli* O157. Nostro et al.^35^ reported that the *T. pubescens* methanolic extract had no antibacterial activity against gram-negative bacteria such as *E. coli*, *P. aeruginosa*, and *Salmonella *spp., while the *T. pubescens* essential oil had very strong inhibitory effects against such bacteria, even in diluted forms. Among the most important components of *T. syriacus*, carvacrol (MIC_90_: from <0.375 to 6.25 µl/ml) and thymol (MIC_90_: from <0.375 to 1.5 µl/ml) exhibited the best inhibitory activities against the tested gram-negative isolates.^36^

It is worthy of note that the essential oil antimicrobial activity in the present study was associated with the concentration of thymol and carvacrol chemotypes. Our results chime in with those reported by Burt concerning the activity of carvacrol against *E. coli* (MIC range=0.225-5 μl/ml), but not for the activity of thymol (MIC range=0.225-0.45 μl/ml).^21^ Similar to our results, Figueiredo et al.^37^ found that the *T. capitata *essential oil, which is rich with carvacrol, was effective against *Salmonella* spp. and *E. coli*.^37^ De Martino et al.^38^ reported that essential oil components, particularly phenols such as carvacrol and thymol, had good antimicrobial activity effects.

## Conclusion

The *T. syriacus* essential oil and its components exhibited very good inhibitory effects against some Syrian gram-negative isolates in the present study. The most effective components were thymol, carvacrol, dihydro-carvon, and linalool, respectively.

We recommend that the synergistic and antagonistic effects of these components be further tested in future clinical trials. 
